# Patterns of taxonomic, phylogenetic diversity during a long-term succession of forest on the Loess Plateau, China: insights into assembly process

**DOI:** 10.1038/srep27087

**Published:** 2016-06-08

**Authors:** Yongfu Chai, Ming Yue, Xiao Liu, Yaoxin Guo, Mao Wang, Jinshi Xu, Chenguang Zhang, Yu Chen, Lixia Zhang, Ruichang Zhang

**Affiliations:** 1 Key Laboratory of Resource Biology and Biotechnology in Western China, Northwest University, Taibai north Rd.229, Xi’an City, Shaanxi Province, China; 2 Plant Ecology Department, University of Tuebingen, Auf der Morgenstelle 3, 72076 Tuebingen, Germany

## Abstract

Quantifying the drivers underlying the distribution of biodiversity during succession is a critical issue in ecology and conservation, and also can provide insights into the mechanisms of community assembly. Ninety plots were established in the Loess Plateau region of northern Shaanxi in China. The taxonomic and phylogenetic (alpha and beta) diversity were quantified within six succession stages. Null models were used to test whether phylogenetic distance observed differed from random expectations. Taxonomic beta diversity did not show a regular pattern, while phylogenetic beta diversity decreased throughout succession. The shrub stage occurred as a transition from phylogenetic overdispersion to clustering either for NRI (Net Relatedness Index) or betaNRI. The betaNTI (Nearest Taxon Index) values for early stages were on average phylogenetically random, but for the betaNRI analyses, these stages were phylogenetically overdispersed. Assembly of woody plants differed from that of herbaceous plants during late community succession. We suggest that deterministic and stochastic processes respectively play a role in different aspects of community phylogenetic structure for early succession stage, and that community composition of late succession stage is governed by a deterministic process. In conclusion, the long-lasting evolutionary imprints on the present-day composition of communities arrayed along the succession gradient.

Global environmental changes and anthropogenic disturbance are increasingly affecting plant biodiversity and ecosystem functioning at both regional and local scale^1^,^2^,^3^,^4^,^5^. Quantifying the drivers underlying the spatial distribution of biodiversity within local communities is a critical issue in ecology and conservation^6^, and also can provide insights into the mechanisms of community assembly^7^,^8^.

There are two major hypotheses proposed to explain contemporary distribution of species diversity^9^, niche-based deterministic and neutrality-based stochastic hypotheses. Niche-based theories predict that factors such as biotic filtering (e.g. competition, facilitation and predation) and abiotic filtering (environmental conditions) play a primary role in structuring species assemblages in local communities^10^,^11^. In contrast, neutrality-based theories emphasize that functional differences between species are unimportant and communities are neutrally or stochastically assembled by probabilistic dispersal, ecological drift or historical inertia^12^. Many studies have shown that both deterministic and stochastic processes play a role in resulting in species co-occurrence patterns but that their relative importance depends on prevailing environmental conditions^13^,^14^,^15^.

Succession can be viewed as a community assembly in progress^16^ and has served as a lens to understand how ecological communities are assembled^17^,^18^. To be able to predict ecosystem responses to future disturbance events and environmental changes, we need a better understanding of the processes that govern community assembly, and thus generate biodiversity, during succession^19^. Theory predicts that, as succession proceeds, the relative importance of abiotic and biotic filtering processes is likely to change^20^. Species in newly opened areas experience environmental adversity, thus environmental and dispersal filtering are likely to structure early stage development^21^. As species accumulate, environmental adversity is alleviated, and biotic filtering increasingly dominates later stages of succession^22^. Earlier studies of plant community assembly during succession mainly focused on temporal changes in taxonomic (species) composition, on changes in single traits or on changes in functional groups for herbaceous or woody plant communities, respectively^23^,^24^. However, a purely taxon-based approach or single traits-based approach cannot take into account ecological differences between species, because the evolutionary history underlying the distribution patterns is often ignored^25^. This limitation may result in biased conclusions about how biodiversity is distributed along succession gradients and the processes underlying community assembly^26^,^27^. To gain an insight into the extent to which the processes governing community assembly during secondary ecosystem development change over time, there is a need for studies of succession that include different facets of diversity within as well as between stages. Phylogenetically based analyses appear to be a valuable approach to test the relative importance of the evolutionary imprint on present-day patterns of coexistence^28^,^29^. This approach connects the evolutionary history of coexisting organisms with ecological mechanisms driving patterns of distribution and abundance^30^,^8^,^31^. Although, phylogenetic diversity does not reflect the diversity of phylogenetically conserved traits^32^,^33^, it is still a primary part accounting biodiversity, and often used as a proxy for functional trait diversity^29^,^34^, as it potentially integrates a greater amount of trait information than is provided by a limited set of measurable traits. Examining the phylogenetic distribution of species in the context of forest succession could further refine mechanistic hypotheses on species coexistence^35^,^36^,^37^ and growth-mortality trade-off accounting for life history differences among species from different succession stages^38^.

Diversity could be partitioned into within- (alpha) and between- (beta) community components based on species or phylogenetic differences. Phylogenetic beta diversity measures phylogenetic distances among communities in a phylogenetic framework. Research of phylogenetic beta diversity addresses the question of how ecological and evolutionary factors interact to influence variations in species compositions of communities across a spatial extent or along a succession gradient. If filtering processes plays a primary role in determining the difference in species composition between local communities, there would be not only a non-random phylogenetic structure within a local community but also a non-random phylogenetic structure in the turnover of species between local communities^39^,^40^. In other words, patterns of within-assemblage phylogenetic structure would ultimately lead to patterns in phylogenetic turnover between assemblages^41^,^42^.

A few recent studies^43^,^44^,^45^,^46^,^37^ have compared temporal changes of plant species or phylogenetic diversity and phylogenetic relatedness among species within communities during succession and tested the extent to which stochastic and filtering processes drive community assembly, but contrasting patterns have emerged. Studies of changes in phylogenetic alpha diversity during tropical forest succession found that later successional communities contained more-distantly related species than early successional communities^31^,^47^,^48^,^49^. In contrast, Letten *et al.* found that communities became more phylogenetically and functionally clustered with time after fire^50^. A study of phylogenetic and functional beta diversity of tropical tree communities showed that phylogenetic turnover between successional stages was random^51^. Additionally, all these studies only focus on herbaceous or woody plant communities separately and have rarely involve a long-term succession of forest (from herbaceous community to forest climax community) and never involve Loess plateau forest landscapes characterized by the alternation of summer drought stress and winter cold stress. Previous studies have shown that patterns of diversity for woody plants often differ substantially from those for herbaceous plants^52^. Large woody plants generally have climate-dominated niches, whereas herbaceous plants have edaphic and microhabitat-dominated niches. Accordingly, it is very worthwhile to analyze the process of community assembly for woody and herbaceous plants of forests simultaneously.

In the present study, we assessed taxonomic, phylogenetic (alpha and beta) diversity at different successional stages within a chronosequence representing a more than 200-year-long succession, across whole 6 successional stages from abandoned field to climax forest on the Loess Plateau of northern China. The climate is a semi-arid temperate continental monsoon climate^53^. Changes in climate and anthropogenic interference led to the degradation of natural vegetations and wide areas of vegetations begin to be restored after conservation^54^. In this region, conservation and restoration of vegetation are acquiring notable importance due to land degradation. Large scale secondary landscapes in this area are characterized by a mosaic of community patches that represent different stages in the succession from annual herb to climax forest stage. The different-staged patches of vegetation are assumed to represent a temporal sequence of change in community composition^55^. This succession series are most suited for the analysis of community characteristics, such as biological diversity, that change over time, and can contribute to an understanding of processes of community assembly.

We used this data set to address the following questions: (1) whether there are contrasting changes in taxonomic and phylogenetic diversity within communities (alpha diversity), together with the taxonomic and phylogenetic turnover between communities (beta diversity), at six succession stages during a long-term succession. (2) we examined, for each successional stage, whether species co-occurring within stage is phylogenetically more (or less) similar than expected. If vegetation composition during succession is a turnover of dominant assembly mechanism, is shrub the key stage of transition. (3) Whether herbaceous and woody plants of communities showed different assembly process during the course of late succession. The fulfillment of this study may provide new evidence of transitional assembly process in warm temperate forest zone and has a bearing on community assembly theory, and will simultaneously provide application basis to guide vegetation restoration and reconstruction in region of the Loess Plateau.

## Material and Methods

### Study site

The study was performed in the Ziwuling region (N35°09′–35°40′, E108°47′–108°57′) located in the middle of the Loess Plateau, Shaanxi, China. Elevation ranges from 1100 m to 1150 m. The climate is a semi-arid temperate continental monsoon climate, with generally frequent heavy rainfall events in summer. Mean annual precipitation is 550–650 mm and mean annual temperature is around 9–11 °C. The research area is characterized by the integrated chronosequence of secondary forests, from abandoned agricultural fields to mature forests. Vegetation was surveyed between June and September in 2011 and 2012. A set of 90 plots were established in the study area, comprising 63 20 m × 20 m plots for woody dominated communities (stage 4–6) and 27 10 m × 10 m plots for herbaceous dominated communities (stage 1–3) (Table 1). All species within each plot were identified, and abundance, coverage, height and life forms (woody vs. herbaceous) of the species were documented. All the plots were assigned to six succession stages represented by Zhu^53^. Specifically, stage one (1–4 yr) is dominated by annuals, while stage two (4–8 yr) is dominated by herbaceous perennials, *Artemisia gmelinii* and *Artemisia sacrorum* (Compositae). Stage three (8–15 yr) is dominated by perennial grass (Gramineae) and stage four (15–50 yr) is a shrub community. Up to stage five (50–100 yr), pioneer trees species become the prominent growth form. Finally, species from the genus *Quercus* dominate the climax forest stage (>100 yr).

### Diversity measures

#### Taxonomic alpha and beta diversity

Taxonomic alpha and beta diversity were characterized by Simpson diversity index^56^ and the 1-Jaccard index^57^ respectively.

#### Phylogenetic alpha diversity

A phylogeny for all species found in the 90 plots was obtained by using the informatics tool Phylomatic^58^ (available at http://www.phylodiversity.net). Phylomatic uses the Angiosperm Phylogeny Group’s APGIII consensus tree (R20120829) as a backbone onto which species are added based on their taxonomy. Branch lengths for each tree species were estimated using the BLADJ algorithm^59^, and node dates were estimated from Wikstrom *et al.*^60^. We used Faith’s phylogenetic diversity (PD) metric^61^ to quantify the phylogenetic alpha diversity of each plot. Faith’s PD has the advantage of being phylogenetic diversity metric in conservation research^62^,^63^.

We used the net relatedness index (NRI) and Nearest Taxon Index (NTI)^28^ to quantify the degree of phylogenetic relatedness among species within each plot. These metrics were estimated with the COMSTRUCT algorithm^29^ implemented in Phylocom. NRI measures the standardized effect size of the mean phylogenetic distance (MPD), which estimates the average phylogenetic relatedness between all possible pairs of taxa in a community. The NTI calculates the mean nearest phylogenetic neighbor among the individuals in a community. Random communities were generated by drawing species from phylogeny pool, while maintaining per-plot species richness and the frequency of species occurrence among plots. The species pool used in these randomizations included all the species occurring in the study region. NRI and NTI are defined as follows:


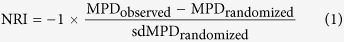



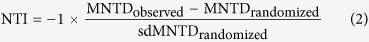


where MNTD/MPD_observed_ is the observed MNTD/MPD, MNTD/MPD_randomized_ is the expected MNTD/MPD of the randomized assemblages (n = 999) and sdMNTD/MPD_randomized_ is the standard deviation of the MNTD/MPD for the randomized assemblages. A positive NTI/NRI value indicates that MNTD/MPD is lower than that expected by chance and that phylogenetic clustering of species occurs. Conversely, a negative NTI/NRI value indicates phylogenetic overdispersion^28^.

#### Phylogenetic beta diversity

For each pair of plots within succession stages, we calculated a phylogenetic distance which was considered as a measure of phylogenetic beta diversity. Phylogenetic distances were estimated with the COMDIST algorithm^29^ implemented in Phylocom.

Two metrics betaNRI and betaNTI were estimated with the COMDIST and COMDISTNT algorithm^29^ implemented in Phylocom. These metrics are analogous to the NRI and NTI alpha metrics, where the betaNRI calculates the mean phylogenetic distances for each pair of individuals between two communities. The betaNTI calculates the mean nearest phylogenetic neighbor among the individuals between two communities. As with the alpha metrics NRI and NTI, the beta metrics used the same null model and species pool, and negative values of betaNRI and betaNTI indicate higher-than-expected phylogenetic turnover given the species turnover, meaning that each community generally contains distantly related individuals. Conversely, positive values indicate lower phylogenetic turnover than expected given the species turnover, meaning that turnover between the two communities occurs between closely related individuals.

#### Data analysis

Measures of taxonomic and phylogenetic alpha diversities were calculated for each plot within the six successional stages. Phylogenetic and taxonomic beta diversities were calculated between pairs of plots belonging to the same successional stages. Differences in mean diversity between the six successional stages were assessed with ANOVA and post hoc pairwise comparisons (*Student–Newmans–Keuls*) were performed when required. Additionally, the same analyses were performed separately for woody and herbaceous plants for the late succession stages (stage 4 to stage 6). The species pool used in these analyses included all the species occurring in the study region. All the metrics (NRI, NTI, betaNRI and betaNTI) were calculated by using both abundance and occurrence (presence/absence) data. For abundance-weighted indices, we weighted the pairwise distances among species by their relative coverage. These measures were averaged among plots within each successional stage so that the significance of overall patterns could be assessed by two-tailed t-tests. All the analyses were performed with R software^64^.

## Results

### Changes in taxonomic and phylogenetic diversity within stages during succession

**Figure 1:**
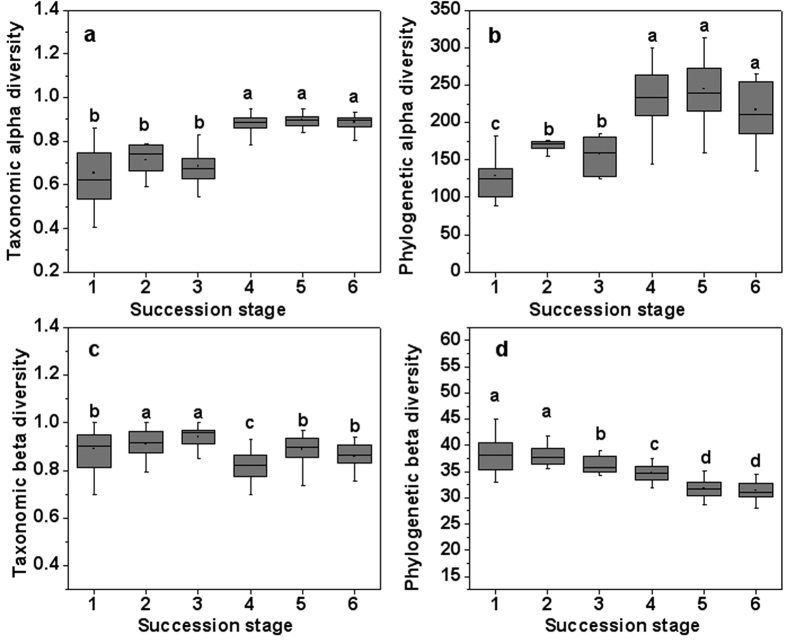
Taxonomic (**a,c**) and phylogenetic (**b,d**) alpha and beta diversity (mean ± SD) within six successional stages. Letters indicate significant differences (α = 0.05) between the stages.

A total number of 356 angiosperm species were found in the 90 plots, of which 129 were woody plants and 227 were herbaceous plants. It was clear that taxonomic and phylogenetic α-diversity showed consistent increasing patterns over succession (Fig. 1a,b). The two facets of between-plot diversity within the succession stages showed absolutely different temporal patterns. Taxonomic beta diversity did not show a regular pattern, while phylogenetic beta diversity decreased throughout succession (Fig. 1c,d).

Stage 4 showed higher taxonomic alpha diversity of herbaceous plants than stage 5 and 6, while it had lower taxonomic alpha diversity of woody plants compared to stage 5 and 6. Phylogenetic alpha diversity of herbaceous plants tended to decrease from stage 4 to 6, while it did not change for woody plants (Fig. S1).

### Null model analysis

**Figure 2:**
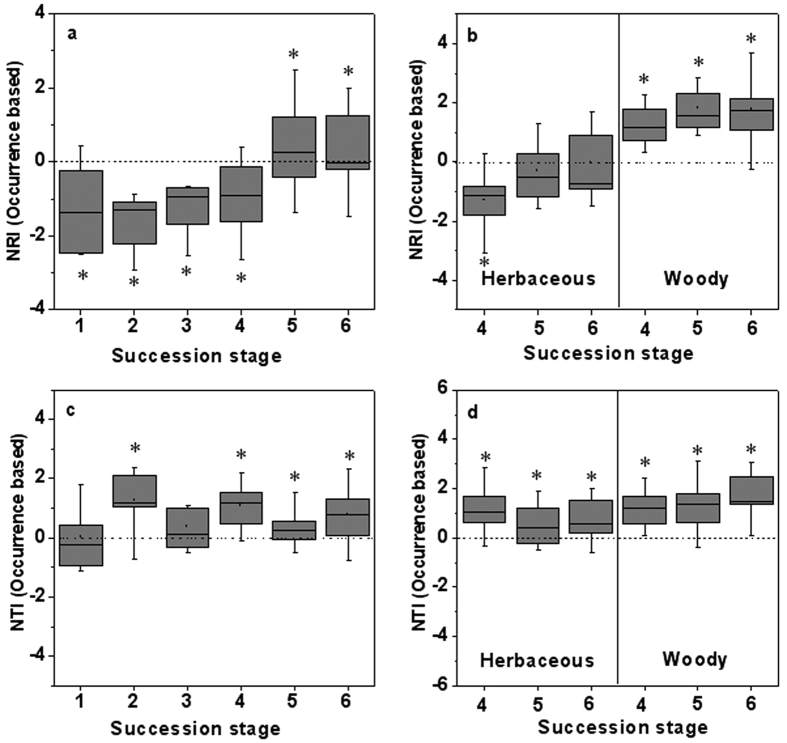
Patterns of Net relatedness index (NRI a,b) and Nearest Taxon Index (NTI c,d). (**a,c**) patterns across six successional stages and (**b,d**) for herbaceous and woody plants in the late three successional stages. Specifically NRI or NTI <0 represents phylogenetic divergence, while NRI or NTI >0 indicates phylogenetic convergence. Asterisks indicate overall significance according to two-tailed *t*-tests (p < 0.05). See Methods for details.

The Net Relatedness Index (NRI) within the succession stages increased during succession (Fig. 2a), and communities transitioned from phylogenetic overdispersion to clustering. Temporal patterns of NRI based on occurrence were congruent with that based on abundance measures (Figs 2 and S2), although no significant patterns were detected for NRI values based on abundance at stage 1, stage 4 and stage5 (Fig. S2). In addition, the NRI and NTI results were not consistent (Fig. 2). The NTI values were on average more phylogenetically clustered or random for early succession stages (Fig. 2a), but for the NRI analyses, these stages were phylogenetically overdispersed (Fig. 2c). Woody plant assemblages differed from herbaceous plant assemblages in several aspects for late three succession stages (Fig. 2b,d). The NRI values of herbaceous plant were on average more phylogenetically overdispersed or random, but for the NTI analyses, they were phylogenetically clustered. The NRI and NTI of woody plants within the late three succession stages were all significantly phylogenetically clustered.

**Figure 3:**
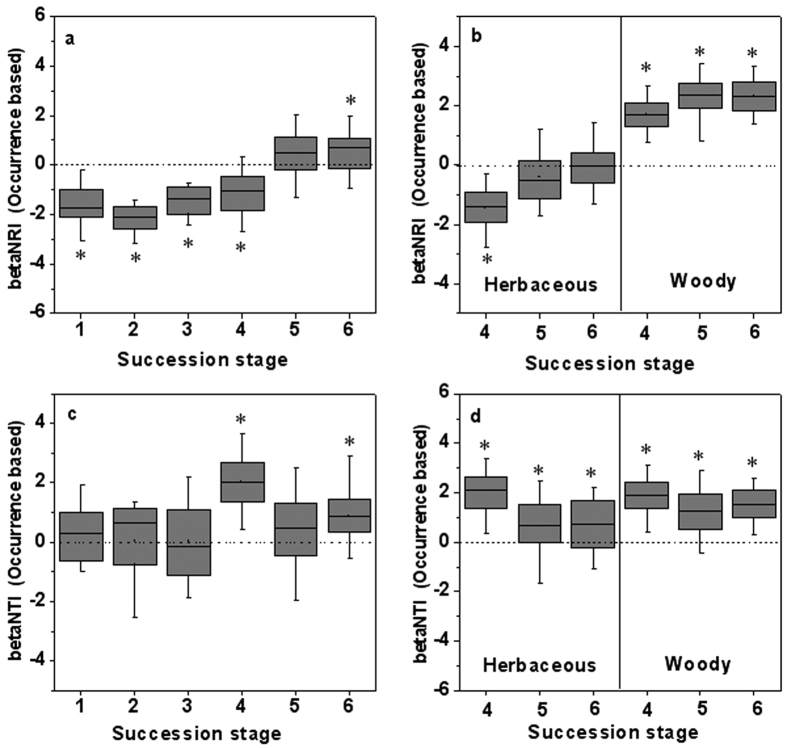
Patterns of beta Net relatedness index (betaNRI a,b) and beta Nearest Taxon Index (betaNTI c,d). (**a,c**) patterns across six successional stages and (**b,d**) for herbaceous and woody plants in the late three successional stages. Specifically betaNRI or betaNTI <0 represents phylogenetic divergence, while betaNRI or betaNTI >0 indicates phylogenetic convergence. Asterisks indicate overall significance according to two-tailed *t*-tests (p < 0.05). See Methods for details.

The betaNRI showed qualitatively the same increasing trends with NRI during succession (Fig. 3a), indicating that phylogenetic turnover between the communities occurs between distantly related individuals at the early stages, while occurs between closely related individuals at the final stages. Moreover, betaNRI was significantly greater or less than zero for all stages except for stage 5 (Fig. 3a). The temporal patterns of betaNRI based on occurrence and abundance measures were consistent (Figs 3 and S3), although no significant patterns were detected for betaNRI values based on abundance at stage 1, stage 3 and stage 4 (Fig. S3a). BetaNTI values did not show a regular pattern with succession either based on occurrence or abundance measures (Figs 3c and S3c). The betaNTI values for early stages were on average more phylogenetically random, but for the betaNRI analyses, these stages were phylogenetically overdispersed (Fig. 3c). For the late three succession stages, the betaNRI values of herbaceous plants were on average more phylogenetically overdispersed or random, but for the betaNTI analyses, they were phylogenetically clustered (Figs 3b,d and S3b,d). BetaNTI and betaNRI of woody plants within the late three succession stages are all significantly higher than zero (Figs 3b,d and S3b,d).

## Discussion

### Phylogenetic and taxonomic patterns of alpha diversity

Understanding the pathways and endpoints of recovery is not only essential for restoration but also to predict how plant communities respond to environmental change^19^. In the present research, both taxonomic and phylogenetic alpha diversity increased during succession. This is consistent with previous studies which found that taxonomic and phylogenetic diversity increased with successional age either in forest^46^,^65^ or in herbaceous plant communities^20^. Interesting, we also found that although the phylogenetic alpha diversity significantly increased with succession, communities of later stages became more phylogenetic clustering. This pattern was jointly promoted by recruitment and mortality processes. Increasing phylogenetic alpha diversity was caused by the recruitment of more species as succession process^66^, while later communities selected the colonists that are similar to the residents during this recruitment process. Generally, a severe environment is more likely to lead to phylogenetic clustering^50^. In the Loess Plateau and other semi-arid ecosystems, water is a major factor limiting plant growth^54^, and the limiting effect may increase as the number of woody plant increases during succession and contributes to the selection of colonists for late succession stages. We also recorded an increased number of coniferous and barbed plants along the succession series, which suggests an increasing deficit of water supply. However, according to a classical axiom of community ecology, the relative importance of biotic processes (e.g. competition) may increase as communities mature and then lead to phylogenetic overdispersion^67^. Therefore, different ecological processes, such as competition exclusion and environmental filtering, may work together and produce this pattern during succession.

Based on NRI, most recent studies found increasing phylogenetic overdispersion as succession proceeds. However, we identified an overall shift from phylogenetic overdispersion to clustering and tested our hypothesis that shrub stage is a transition from phylogenetic overdispersion to clustering. This pattern of phylogenetic structure is also consistent with the study of heathland succession after a fire^50^. In the Loess Plateau, natural vegetations experienced serious anthropogenic interference, which is similar to a fire, before conservation and restoration of vegetation^54^. Stage 2 and stage 3 are dominated by perennial *Artemisia* and grass species that generally could produce more seeds to enhance germination and establishment in an annual herbaceous community^53^, competitive exclusion of closely related species and/or colonisation of distantly related species drive phylogenetic overdispersion. The shrub stage is a transition from grass communities to forests. After the perennial grass stage, most of the grasslands are replaced by shrub communities. The woody encroachment leads to the loss of original species and evolutionary history^68^, generating an in-between phylogenetic pattern from grass to forests. NRI values of the stage 5 and 6 were higher than zero, indicating phylogenetic clustering. First, more woody plants in later communities are associated with high competition for solar radiation and water representing strong biotic filters. Such demanding conditions are expected to filter out many lineages not adapted to such habitat types, leaving those that can tolerate the abiotic template to result in convergent adaptation. For example, a recent research has also reported that plant clades with particular adaptations to dry forest habitats are the result of recent evolutionary radiations, which would lead to patterns of phylogenetic clustering^69^. An alternative hypothesis is that some species already adapted to these conditions occupy late stages in the study system^70^. In the present study, Quercus is the common and dominant genera in the climax communities and represent the contribution of completely novel lineages to the late community assemblages. Both the hypotheses described above may be responsible for the phylogenetic pattern of late succession communities.

Specifically, we also found that the NTI and NRI results were not consistent. The NTI values of early succession stages were on average more phylogenetically clustered or random, but for the NRI analyses, these stages were phylogenetically overdispersed. The net relatedness index (NRI) mainly measures relatedness across the community. Yet, the nearest taxon index (NTI), which measures distances to the closest relative, is expected to be the more powerful statistic for detecting limiting similarity^30^. This suggests that limiting similarity may not be the major factor driving overdispersion of early succession stages. However, the relative power of NTI and NRI varies as a function of multiple variables, making it difficult to specify why results differed between the two statistics^30^,^35^. The NRI and NTI based on species abundances showed inconspicuous but similar phylogenetic trend over time compared to that based on occurrence, suggesting a weak impact of species abundance on the phylogenetic structure of the community.

### Phylogenetic and taxonomic patterns of beta diversity

The two facets of beta diversity within the succession stages showed absolutely different temporal patterns. Taxonomic beta diversity did not show a directed pattern, while phylogenetic beta diversity decreased throughout succession. The assembly and maintenance of ecological communities reflect the net sum of many ecological processes that often act on species similarities and differences^18^. These similarities and differences are mainly about the function and relatedness of species. Environmental conditions might select special species based on phylogenetic and functional similarities or differences, rather than species itself. Besides, phylogenetic diversity reflects the evolutionary history of a community, which may also reflect its functional diversity^29^,^34^, as it potentially integrates a greater amount of trait information. Therefore, environmental heterogeneity only acted at phylogenetic level rather than taxonomic level.

The betaNRI showed increasing trends during succession, indicating that phylogenetic turnover between the communities occurs between distantly related individuals at the early stages, while occurs between closely related individuals at the final stages. The pattern of phylogenetic turnover within successional stages may be explained by the effects of strong environmental or/and biotic filtering^71^,^72^. Plots with lower differences in environmental condition have lower phylogenetic compositional turnover, whereas plots with higher differences in environmental condition exhibit higher phylogenetic turnover^8^,^73^. Indeed, humans disproportionately disturbed specific areas of the Loess plateau^53^. Therefore, the species in newly opened areas experience strong environmental heterogeneity which leads early succession stages showing higher phylogenetic turnover. As the accumulation of species number and the development of soil, the environmental heterogeneity limiting species movements to other environmental ranges is alleviated and then species of the late succession stages tend to recover their ancestral environmental distributions^74^,^75^. At the same time, the biotic interactions such as competition increasingly may also work in the phylogenetic structure of later succession stages^21^. Because, if most species are competitively excluded from the community, the remaining assemblages should show a low value of phylogenetic beta diversity since the common dominant species is present in most plots. Although most pairwise plots for stage 5 shown phylogenetic clustering (betaNRI >0), the mean betaNRI for this stage did not show significant difference from zero. This is consistent with the result of a study on tropical forest^76^, which indicates that phylogenetically conserved traits may not play a large role in governing the species composition of this stage. Alternatively, different deterministic processes, such as competition exclusion and environmental filtering, may work together, counteract each other, and then produce random MPD values of stage 5^67^.

The relative importance of stochastic versus deterministic processes could be distinguished by null model approach on β-diversity^77^. We found that the betaNTI and betaNRI results were not consistent for early succession stages. The betaNTI values were on average more phylogenetically random for early succession stages, but for the betaNRI analyses, these stages were phylogenetically overdispersed. Together, the results of betaNRI suggest that community composition is initially and finally governed by two opposite deterministic processes, respectively, and the interaction of the two processes may lead to the stochastic patterns at mid-succession stage (stage 5). In contrast, the pattern of betaNTI showed a shift from early stochastic process to late deterministic process. This shift is consistent with a role for recent local diversification in determining community structure, as betaNTI reflects shallow (recent) phylogenetic structure^78^. Over all, we suggest that deterministic and stochastic processes play a role in different aspects of community phylogenetic structure for early succession stage and that community composition of late succession stage is governed by a deterministic process.

### Herbaceous and woody plants showed different assembly process

Generally, for forest and shrub communities, the canopy harbors more species and greater phylogenetic diversity than the understory, and this is interpreted as that multiple seedling cohorts are recruited into the canopy during succession^79^. Moreover, the recruitment during the later phases of succession favors more phylogenetically distant taxa than during early succession due to density dependent mortality^80^. Our comparison of woody plants from 4–6 stages did not support this interpretation, because species mean phylogenetic distance did not change significantly with community age.

Of the 63 forest and shrub plots from late three succession stages, most plots had positive values of NRI/NTI and betaNRI/betaNTI for woody species, indicating that phylogenetic clustering dominated in woody assemblages. Non-random patterns of woody plant co-occurrence in forests are well-documented^35^,^46^. For the forest in Borneo, an overall pattern of phylogenetic clustering was detected^28^, which is consistent with our results. To the contrary, the coexisting woody species in secondary tropical forests of Costa Rican were more distantly related than expected by chance^46^. Overall differences between these patterns may be attributed to several factors. Firstly, the different environment conditions may contribute to this. A previous study has showed that water availability may underlie species sorting in dry forest assembly and reduce functional overdispersion^21^. In the Loess Plateau, water is a major factor limiting plant growth^54^, especially for woody species. This limiting effect may reduce the phylogenetic overdispersion. But for tropical forests, it is not the case. Additionally, the higher intensity of disturbance in the Loess Plateau might also affect the successional trajectory in this region. Because phylogenetic clustering of woody plant assemblages were generally found in these past disturbed regions, such as grazing, fire and anthropogenicmanagement^50^. The NRI and betaNRI values of herbaceous plant were on average more phylogenetically overdispersed or random, but for the NTI and betaNTI analyses, they were phylogenetically clustered. These results suggest that deterministic and stochastic processes play a role in different aspects of phylogenetic structure of herbaceous plant; increased competition does not necessarily lead to increased phylogenetic overdispersion.

### A caution of method and an implication for ecological restoration

The use of space-for-time substitutions in chronosequences is common in ecological studies aimed at understanding long-term and strongly directional dynamics, while this method assumes that spatial and temporal variation are equivalent^81^,^82^. We do not have direct temporal data of colonization and extinctions along the succession; however, most of herbaceous dominated communities converted to pine- and oak-dominated woodlands during the twentieth century^53^ and species composition of communities from different succession stages now was consistent with previous study^53^, suggesting that sites of different ages are following the same trajectory. Furthermore, the design of comparing directly adjacent habitats was used to minimize the factors other than succession that may have contributed to the compositional differences between successional stages. A limitation of using phylogenies in community ecology is that potential species ecological differences are proportional to the amount of time since they diverged from a common ancestor^83^, closely related species are ecologically similar to each other and functional traits are “conserved”. However, phylogenetic and functional distance is not the proxy of each other^83^. Phylogenies and traits represent different aspects of species’ ecology^83^. Therefore, phylogenetic patterns in this study may only represent functional information of conserved traits rather than convergence traits. One way forward is to integrate phylogeny and traits to investigate whether the phylogenetic changes we observed correspond to changes in functional representation or diversity.

As ecosystems worldwide are degraded by human activity, ecological restoration plays an essential role in maintaining biodiversity and critical ecosystem functions^84^. An essential component of restoration is there assembly of plant communities following ecosystem degradation. A frequent method of community restoration is to re-create the patterns of plant species richness found in remnant vegetation or to conduct a forestation alone, sometimes even introduce exotic species. Our study highlights the fact that a long-lasting evolutionary imprints on the present-day composition of plant assemblages arrayed along the succession gradient. To retain species richness and natural assembly mechanisms during succession, which are of high conservation interest^85^, we proposed that relationships between introduced and native species should be at least partially considered.

## Conclusions

Comparative analysis of taxonomic and phylogenetic diversity within different stages of succession provides insights into the temporal dynamics of the processes that drive post-disturbance biodiversity changes. The changes in phylogenetic diversity during succession differed from those shown by taxonomic diversity suggests that assessments of biodiversity change after disturbance may be misleading if based on a single facet of diversity. Phylogenetic clustering dominates in later communities, whereas multiple patterns co-occur in early communities, indicating that deterministic and stochastic processes play a role in different aspects of community phylogenetic structure for early succession stage and community composition of late succession stage is governed by a deterministic process. Overall, at the scale of our study, the long-lasting evolutionary imprints on the present-day composition of plant assemblages arrayed along the succession gradient.

## Additional Information

**How to cite this article**: Chai, Y.F. *et al.* Patterns of taxonomic, phylogenetic diversity during a long-term succession of forest on the Loess Plateau,China: insights into assembly process. *Sci. Rep.* **6**, 27087; doi: 10.1038/srep27087 (2016).

## Supplementary Material

Supplementary Information

## Figures and Tables

**Table 1:** Succession stages and numbers of plots and species (Yue).

Succession status	Succession status	Number of plots	Number of species
Stage 1	Annual herb stage	9	53
Stage 2	Perennial *Artemisia* stage	9	72
Stage 3	Perennial grass stage	9	63
Stage 4	Shrub stage	37	222
Stage 5	Pioneer forest stage	16	207
Stage 6	Climax forest stage	10	135
