# Supplementary material for: Patterns of taxonomic, phylogenetic diversity during a long-term succession of forest on the Loess Plateau, China: insights into assembly process
Source: Sci Rep. 2016 Jun 8;6:27087. doi: 10.1038/srep27087 (PMC4897607; doi:10.1038/srep27087)
Supplement: Supplementary Information [file srep27087-s1.doc]

**Patterns of taxonomic, phylogenetic diversity during a long-term succession of forest on the Loess Plateau, China: insights into assembly process**

Yongfu Chai^1^, Ming Yue^1^ (corresponding author, yueming@nwu.edu.cn), Xiao Liu^1^, Yaoxin Guo, Mao Wang^1^, Jinshi Xu^1^, Chenguang Zhang^1^, Yu Chen^1^, Lixia Zhang^1^，Ruichang Zhang^2^

**Figure legends**

**Figure S1. Taxonomic (a, c) and phylogenetic (b, d) alpha and beta diversity (mean±SD) for herbaceous and woody plants within the late three successional stages.** Letters indicate significant differences (α= 0.05) between the stages.

**Figure S2. Patterns of Net relatedness index (NRI a, b) and Nearest Taxon Index (NTI c, d) based on abundance weighted.** (a, c) patterns across six successional stages and (b, d) for herbaceous and woody plants in the late three successional stages. Speciﬁcally NRI or NTI <0 represents phylogenetic divergence, while NRI or NTI >0 indicates phylogenetic convergence. Asterisks indicate overall significance according to two-tailed *t*-tests (p<0.05). See Methods for details.

**Figure S3. Patterns of beta Net relatedness index (betaNRI a, b) and beta Nearest Taxon Index (betaNTI c, d) based on abundance weighted.** (a, c) patterns across six successional stages and (b, d) for herbaceous and woody plants in the late three successional stages. Speciﬁcally betaNRI or betaNTI <0 represents phylogenetic divergence, while betaNRI or betaNTI >0 indicates phylogenetic convergence. Asterisks indicate overall significance according to two-tailed *t*-tests (p<0.05). See Methods for details.

**Figure S1(Yue)**

**Figure S2(Yue)**

**Figure S3(Yue)**
